# Supplementary figures and images for: Hyperbaric oxygen therapy improves symptoms, brain’s microstructure and functionality in veterans with treatment resistant post-traumatic stress disorder: A prospective, randomized, controlled trial
Source: PLoS One. 2022 Feb 22;17(2):e0264161. doi: 10.1371/journal.pone.0264161 (PMC8863239; doi:10.1371/journal.pone.0264161)

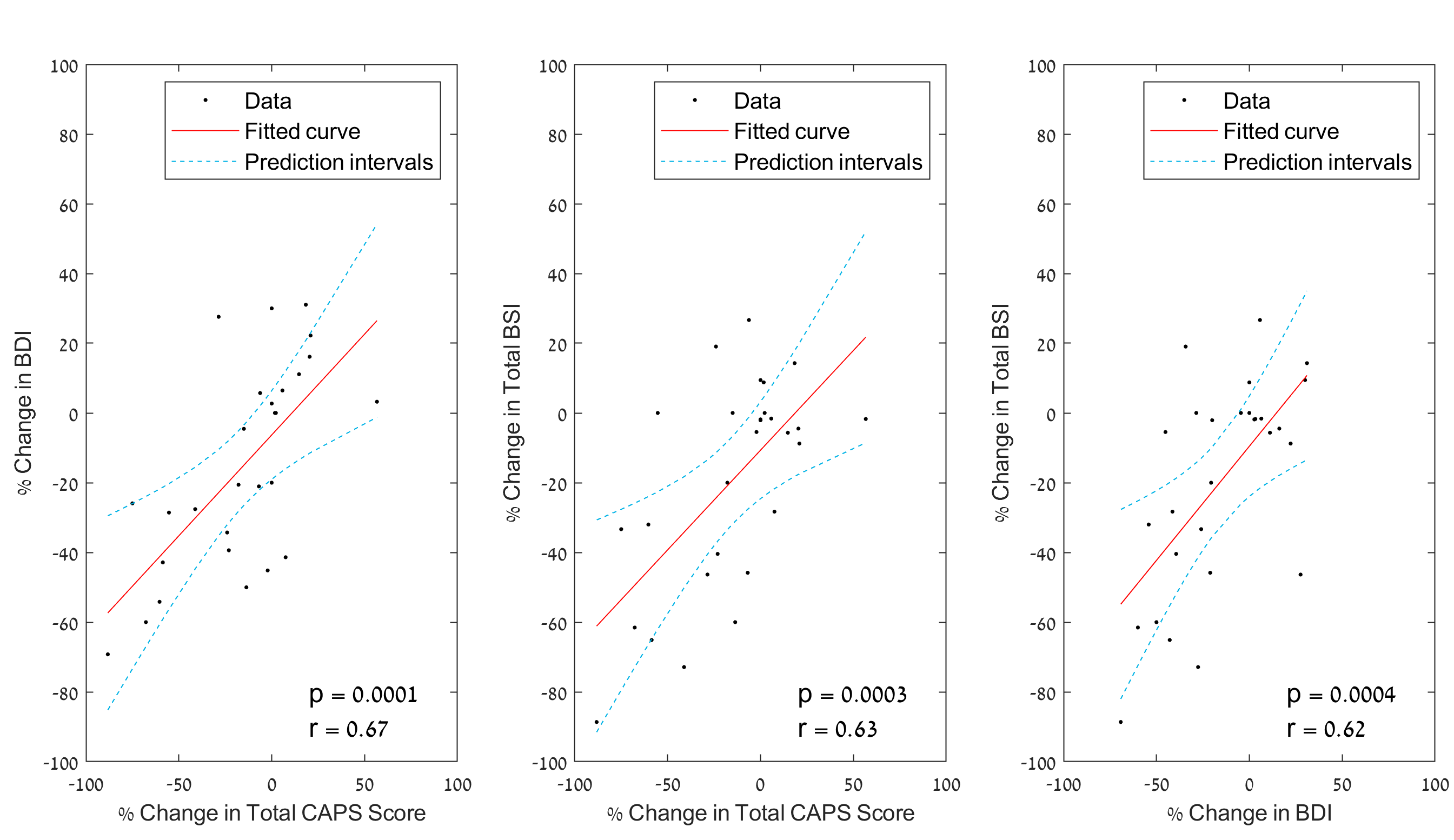

Supplement: S1 Fig — r is Pearson’s correlation coefficient, p < 0.0004 for all comparisons. (TIF) [file pone.0264161.s002.tif]

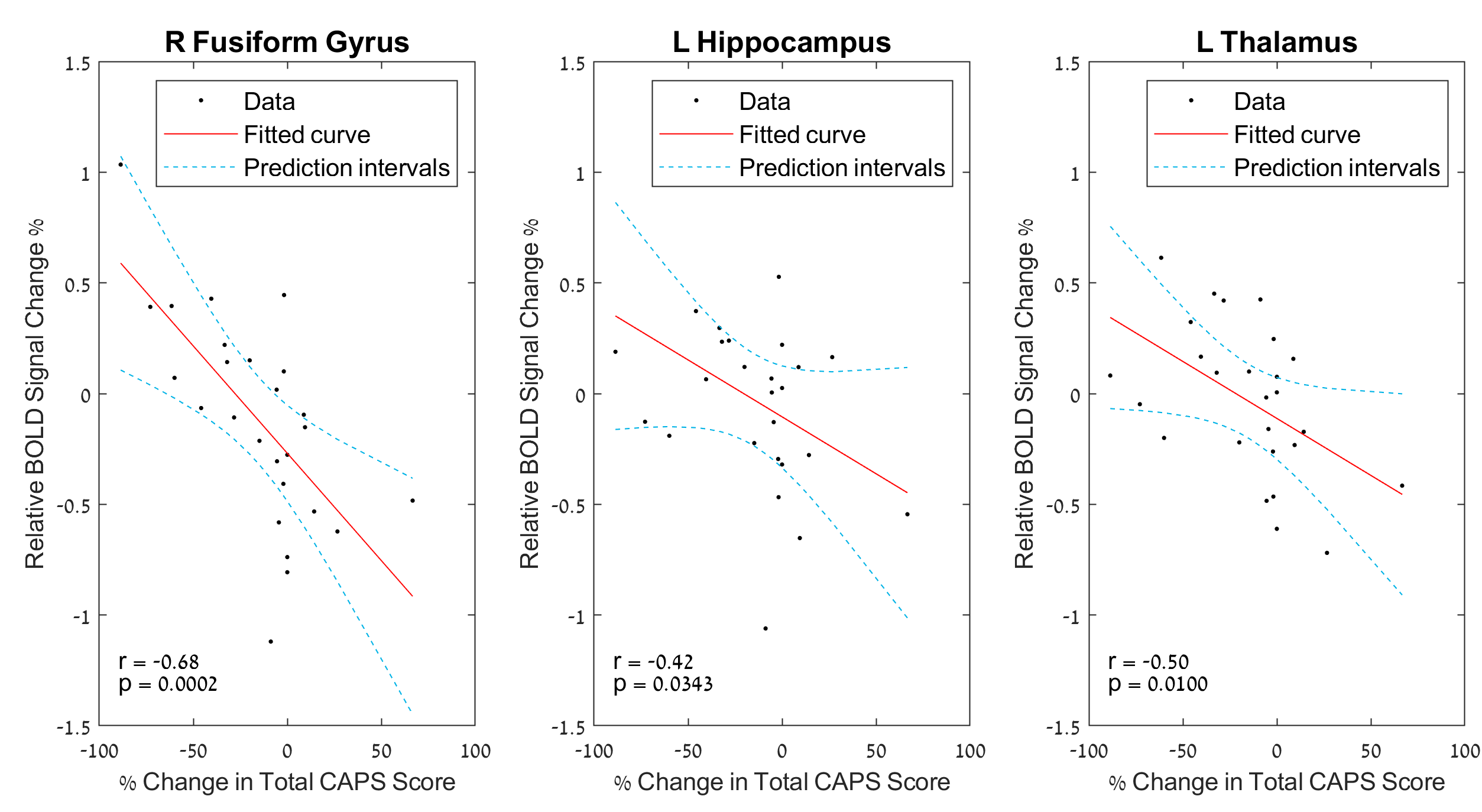

Supplement: S2 Fig — r is Pearson’s correlation coefficient, p < 0.05 for all comparisons. (TIF) [file pone.0264161.s003.tif]

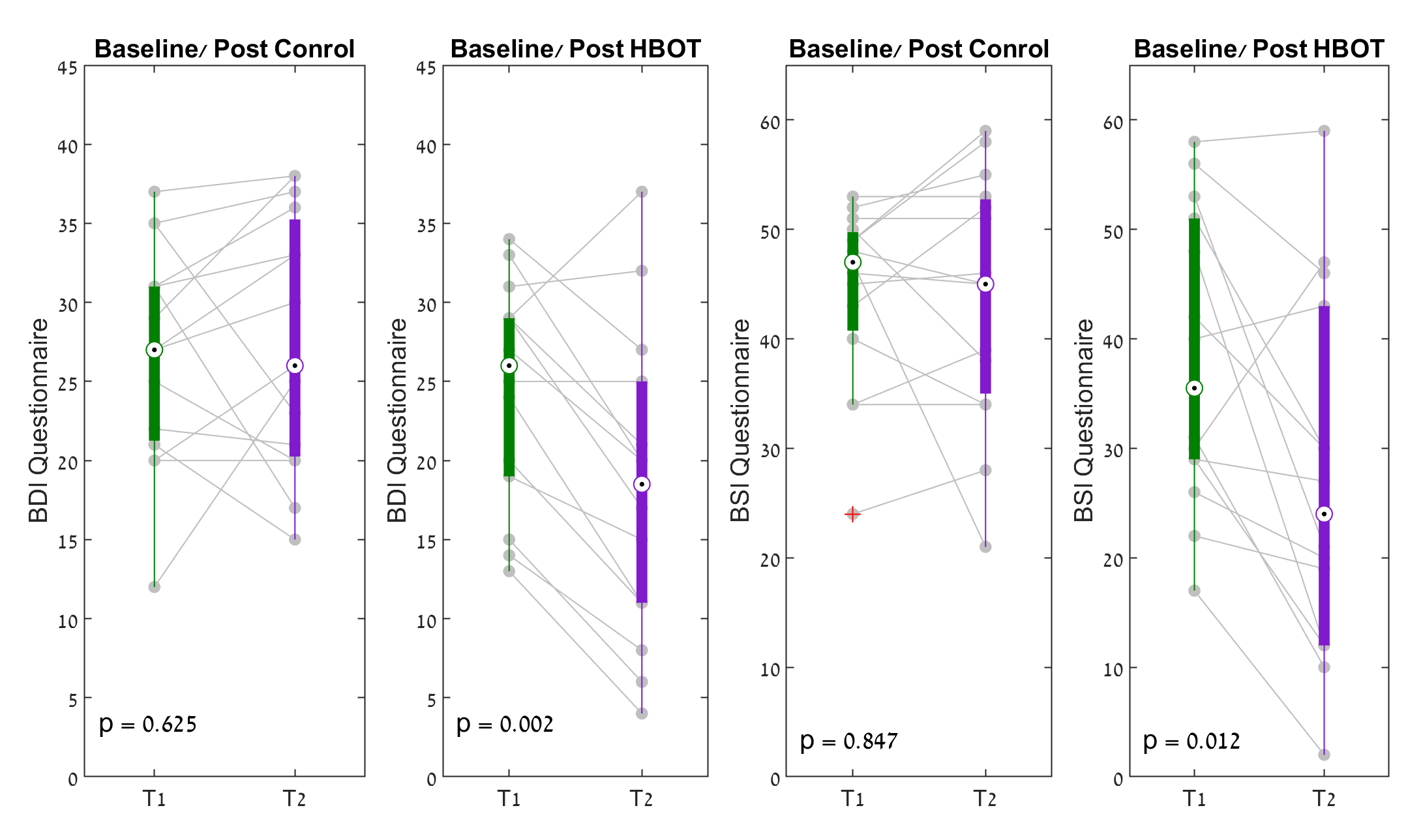

Supplement: S3 Fig — The central mark indicates the median, and the bottom and top edges of the box indicate the 25th and 75th percentiles, respectively. + Symbols indicate outliers. (TIF) [file pone.0264161.s004.tif]
