# Supplementary material for: Hyperbaric oxygen therapy improves symptoms, brain’s microstructure and functionality in veterans with treatment resistant post-traumatic stress disorder: A prospective, randomized, controlled trial
Source: PLoS One. 2022 Feb 22;17(2):e0264161. doi: 10.1371/journal.pone.0264161 (PMC8863239; doi:10.1371/journal.pone.0264161)
Supplement: S1 Table — (DOCX) [file pone.0264161.s005.docx]

| **SI – Table 1**: Total CAPS score repeated measures ANOVA | | | | | | |
| --- | --- | --- | --- | --- | --- | --- |
|  | **Main Effect of Group** | | **Main Effect of Time** | | **Interaction Effect (Group-by-Time)** | |
|  |  |  |  |  |  |  |
|  | ***F*** | ***p value*** | ***F*** | ***p value*** | ***F*** | ***p value*** |
| B. Intrusion symptoms | 8.72 | 0.006 | 23.88 | 0.000 | 28.93 | 0.0000 |
| C. Avoidance symptoms | 5.59 | 0.026 | 9.94 | 0.004 | 23.39 | 0.0000 |
| D. Cognitions and mood symptoms | 2.44 | 0.130 | 8.71 | 0.006 | 13.28 | 0.0011 |
| E. Arousal and reactivity symptoms | 10.66 | 0.003 | 3.80 | 0.062 | 8.50 | 0.0071 |
| T. Total | 8.64 | 0.007 | 19.60 | 0.000 | 30.57 | 0.0000 |

Comparison measures by using a two-factor ANOVA, where group is the between-subject variable, and time is the within-subject variable (pre/ post HBOT for the treatment group; pre/ control for the control group).
