# Supplementary material for: Hyperbaric oxygen therapy improves symptoms, brain’s microstructure and functionality in veterans with treatment resistant post-traumatic stress disorder: A prospective, randomized, controlled trial
Source: PLoS One. 2022 Feb 22;17(2):e0264161. doi: 10.1371/journal.pone.0264161 (PMC8863239; doi:10.1371/journal.pone.0264161)
Supplement: S2 Table — (DOCX) [file pone.0264161.s006.docx]

| **SI – Table 2**: Questionnaire repeated measures ANOVA | | | | | | |
| --- | --- | --- | --- | --- | --- | --- |
|  | **Main Effect of Group** | | **Main Effect of Time** | | **Interaction Effect (Group-by-Time)** | |
|  |  |  |  |  |  |  |
|  | ***F*** | ***p value*** | ***F*** | ***p value*** | ***F*** | ***p value*** |
| **Brief Symptoms Inventory - BSI** | |  |  |  |  |  |
| Total | 7.802 | 0.009 | 6.783 | 0.015 | 5.724 | *0.024* |
| Somatization | 5.147 | 0.031 | 2.490 | 0.126 | 1.255 | 0.272 |
| Anxiety | 10.141 | 0.004 | 6.056 | 0.021 | 3.344 | 0.079 |
| Depression | 3.642 | 0.067 | 6.710 | 0.015 | 10.722 | *0.003* |
| **Beck Depression Inventory** | |  |  |  |  |  |
| BECK | 4.715 | 0.039 | 5.213 | 0.031 | 7.646 | *0.010* |

Comparison measures by using a two-factor ANOVA, where group is the between-subject variable, and time is the within-subject variable (pre/ post HBOT for the treatment group; pre/ control for the control group).
