# Supplementary material for: Hyperbaric oxygen therapy improves symptoms, brain’s microstructure and functionality in veterans with treatment resistant post-traumatic stress disorder: A prospective, randomized, controlled trial
Source: PLoS One. 2022 Feb 22;17(2):e0264161. doi: 10.1371/journal.pone.0264161 (PMC8863239; doi:10.1371/journal.pone.0264161)
Supplement: S1 File — (PDF) [file pone.0264161.s008.pdf]

Hyperbaric oxygen therapy for adult onset Post-Traumatic Stress Disorder (PTSD)

Dr. Keren Doenyas-Barak

[1]The Sagol Center for Hyperbaric Medicine and Research at Assaf Harofhe Medical Center Zerifin, Israel.

OVERVIEW 3

BACKGROUND 3

The traumatic event 3

Memory and brain activity at the moment of trauma 3

Consequences of trauma 6

The clinical outcome of a traumatic event or PTSD 7

Hyperbaric oxygen therapy 7

HBOT and PTSD 8

METHODS 9

Study protocol: 9

Study end points: 10

Primary end point 10

Secondary end points 10

1.PTSD questionnaires: 10

2. Fibromyalgia syndrome (FMS) symptoms 10

3. Quality of life questionnaires: 10

4. Cognitive function 10

5. Brain imaging 12

6. Brain Network Activation (BNA™) Analysis 13

7. Physical activity and sleep 15

5.1 Monitoring 24/7 physical activity 15

8. Immune system 15

9. Endocrine system 15

REF 16

Overview

Post-traumatic stress disorder (PTSD) is a brain's long-term imprint of a traumatic event. Even though data regarding the effect of acute trauma on brain's activity/performance is vast, no effective treatment is currently available that can cure PTSD and return patients to their normal life and activity. At our center, we recently performed a trial of young women with post-traumatic stress disorder accompanied by fibromyalgia after childhood sexual abuse. The results were promising regarding both clinical and radiological outcomes. Great promise has also come from trials in patients with traumatic brain injury (TBI) accompanied by PTSD. The effect of hyperbaric oxygen therapy (HBOT) on pure adult-induced PTSD has not been investigated.

This protocol reviews the pathogenesis of PTSD and the rationale for the use of HBOT as a potential treatment.

Background

The traumatic event

One does not have to be a combat soldier, or visit a refugee camp in order to face trauma. Traumatic events can occur anywhere, as part of natural disasters, terrorist attacks, motor vehicle accidents, or even at home in the setting of violence or sexual abuse.

Trauma occurs as a result of a severely distressing event, in which an overwhelming amount of stress exceeds the ability to cope, or to integrate the emotions involved in the experience [1].

Memory and brain activity at the moment of trauma

Memories are formed in various ways. While we need to concentrate hard to remember some facts, emotionally arousing experiences are usually well remembered [2]. Long-term memory involves several brain regions, such as the hippocampus, striatum and amygdala.

Adrenal stress hormones, epinephrine and cortisol, released by emotional arousal, appear to play an important role in the physiologic regulation of experiential memory (Fig 1) [3] [4]:

- Adrenalin can modulate the memory process by its physiologic effect on the basolateral nucleus of the amygdala (BLA).

- Adrenalin does not cross the blood brain barrier (BBB), and its effect is induced by triggering beta receptors in the afferent fibers of the vagal nerve, which project to the nucleus of the solitary tract of the brain stem.
- Noradrenalin secreted from the amygdala triggers the hippocampus, the striatum and other brain regions; this is a key step in the memory consolidation process.
- The effect of adrenalin on BLA can be blunted by blocking beta adrenergic receptors, or triggered by ketamine anesthesia, which can depress and enhance memory retention, respectively [5].
- Glucocorticoid, secreted at the time of stresssogenic events, triggers the amygdala, the hippocampus and the striatum, and facilitates the consolidation of memories.
- Glucocorticoids also attenuate norepinephrine (NE) secretion in response to adrenergic triggering in the hypophysis.

In the case of a severe traumatic experience, a surge in amygdala's noradrenaline may cause acute activation of several brain regions. This may be accompanied by changes in blood flow and metabolism, which damage cell organelles and especially the mitochondria, as well as increasse inflammation and lysosomal function [6].

- Factors that may contribute to the increased release of NE in response to sympathetic system activation include genetic and stress-induced decrements in neuropeptide Y (NPY) [7], which inhibit NE release, as well as a lower number [8] or affinity [9] of alpha-2-adrenergic autoreceptors.
- Patients with a high number of glucocorticoid receptors are predisposed to pathological stress-induced consequences, and low baseline cortisol levels; these contribute to reduced inhibitory effects on adrenergic responses [10].
- Disturbance in sleep cycles may also be associated with reduced cortisol levels; the latter may be associated with increased consolidation of memory.
- Reports of pharmacological interventions suggest a 6-hour window of time after a traumatic event, during which memories might be disrupted before they are consolidated [11]. This window may reflect cellular processes that occur after binding of stress hormones, such as adrenaline and cortisol, to receptors.
- The use of morphine immediately after injury, during resuscitation and early trauma care, was found to be significantly associated with reduced traumatic memory consolidation [12].
- Pro-inflammatory cytokines, endocannabinoids and neurosteroids may also affect memory under stress response in a more complex manner.

Fig 1.

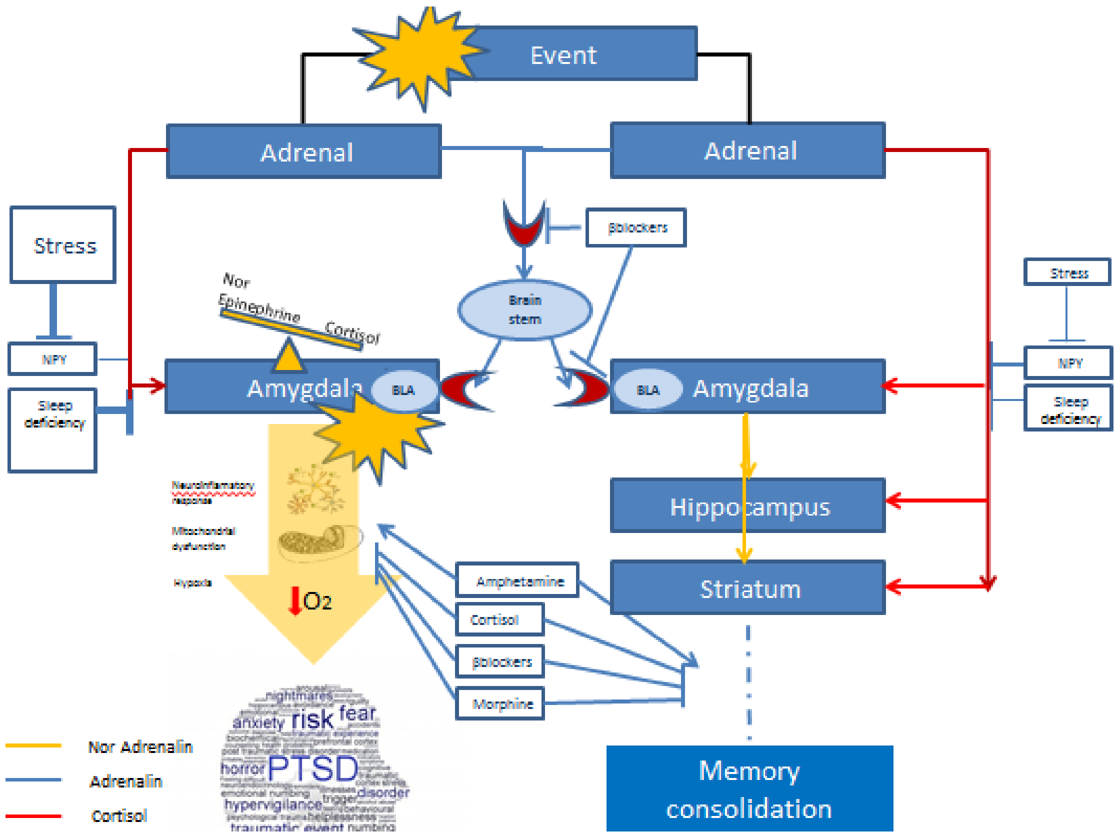

Consequences of trauma

Changes in brain structure and function can be identified months or even years after a traumatic event.
Following the initial phase of elevated stress hormones, an unusual pattern of low (unstimulated) cortisol levels and raised catecholamine levels is evident in trauma survivors [13]. Exaggerated neurochemical and behavioral responses are consistent with central noradrenergic hyper-reactivity [14, 15].

Decreased regional cerebral blood flow (CBF) in several areas, mainly in the right hemisphere, was demonstrated using single-photon emission computed tomography (SPECT)[16, 17] and positron emission tomography (PET) [17].

ASL_MRI also showed significantly decreased rCBF, mainly in the right hemisphere of patients after acute trauma, relative to normal controls. These findings are consistent with PET and SPECT studies in the same population [18].

Fronto-limbic areas are of major interest in the reaction to trauma, and have shown increased activity in some patients, but decreased in others [19]. A small hippocampus was demonstrated after trauma exposure, and related areas such as the medial prefrontal cortex, cingulate gyrus and insula were significantly abnormal in patients after a traumatic experience [20]. Low baseline perfusion to the insula was demonstrated in most trials; however, blood flow to the insula was higher in response to triggers to trauma-related odors in combat veterans exposed to trauma, compared to blood flow in veterans who were not PTSD [21]. Regional CBF changes correlated with, and could be used to predict post-traumatic symptom severity [18]

The clinical outcome of a traumatic event or PTSD

Post-traumatic stress disorder (PTSD) is a clinical condition that develops in up to 30 percent of individuals following exposure to extremely traumatic events. Most individuals who experience trauma react in some degree when experiencing reminders of the trauma. Patients with PTSD, however, experience marked cognitive, affective, and behavioral responses to stimuli, leading to flashbacks, severe anxiety, and fleeing or combative behavior. These individuals compensate for such intense arousal by attempting to avoid experiences that may begin to elicit symptoms; this can result in emotional numbing, diminished interest in everyday activities, and, in the extreme, may result in detachment from relationships [22]. Intrusive symptoms are at the core of the condition, with recurrent memories, nightmares, flashbacks, and psychological reactivity to reminders.

Individuals with PTSD frequently suffer from difficulty sleeping, rapid eye movement (REM) sleep fragmentation and decreased slow wave sleep [23], poor concentration and somatization defined by chronic pain or fibromyalgia. Co-occurring conditions include substance abuse, mood and anxiety disorders, impulsive or dangerous behavior and self-harm [24]. PTSD is also associated with considerable medical comorbidities, including chronic pain and inflammation, cardiometabolic disorders and heightened risk of dementia [25].

Hyperbaric oxygen therapy

A number of clinical studies have presented evidence that hyperbaric oxygen therapy (HBOT) can induce brain repair [26, 27]. Recent trials provide convincing evidence that HBOT-induced cerebral plasticity leads to repair of chronically impaired brain functions and improved quality of life in post-stroke patients and in TBI patients with prolonged post-concussion syndrome, even years after the brain insult. Fibromyalgia,

now recognized as a condition caused by brain malfunction, has also been shown to improve after HBOT- induced neuroplasticity.

Brain insult may result from a variety of brain injuries, which are commonly consequent to impairment of microvascular integrity or cerebral perfusion (whiter regional or global). These may lead to reduced metabolism and neuronal activity, and subsequently to loss of synapses and tampered neuronal connectivity [2,8,9]. The stunned areas are characterized by anaerobic metabolism and ATP depletion, which culminate in stagnation and shortage of energy for the healing processes. This situation may persist for years after the injury [26-32].

HBOT can initiate vascular repair and improve cerebral vascular flow [29], induce regeneration of axonal white matter [33], stimulate axonal growth [34, 35], promote blood–brain barrier integrity and reduce inflammatory reactions, as well as brain edema [36, 37].

HBOT and PTSD

Various animal models provide evidence of potential beneficial effects of HBOT in treating TBI and PTSD [38]. Several trials in patients with TBI have shown great improvement after HBOT [26, 39, 40]. However, these trials did not focus particularly on distinguishing symptoms related to TBI and PTSD. TBI and PTSD are closely correlated, as almost half of the soldiers with mild TBI meet the criteria of PTSD [41, 42].

Only a few trials have focused on PTSD symptoms and the effect of HBOT. Paul G. Harch's study [43, 44] of 16 military persons who received 40 1.5 ATA/60 min HBOT sessions in 30 days, showed less irritability, and fewer nightmares and mood swings in most participants. Generalized Anxiety Disorder 7-item scale and PTSD Checklist-Military (PCL-M) significantly improved, as well.

A recent study performed at our center evaluated the effect of HBOT on symptoms and brain activity in women with fibromyalgia syndrome (FMS) triggered by childhood sexual abuse (CSA) (the data have not yet been published). The study comprised 30 females, aged 26-68 years. Patients were allocated to a treatment or a control group. The HBOT group showed significant improvement in clinically evaluated parameters, compared to the control group. Significant improvement was shown in evaluation of tender points, in the Physical Function Assessment score, and in Quality of Life questionnaires in the HBOT group. Regarding brain imaging, the metabolism in Brodmann's areas 37L, 45L and 8L increased; and in Brodmann's area 25R, 23L, 38L and 28R decreased. Thus, HBOT appears to induce neuroplasticity,

which results in relief of pain and of FMS related symptoms, and improved quality of life for PTSD women with FMS triggered by child sexual abuse.

Study goals

In the proposed study, we intend to evaluate a possible role of HBOT in the treatment of patients with adult-onset PTSD. In addition to the clinical evaluation, the study will include an advanced brain structural and metabolic evaluation.

Methods

The study will include 30 individuals with PTSD as a result of a traumatic event that happened in their adult lives. PTSD will be defined according to DSM-V criteria. Each participant will be evaluated at the time of recruitment by a psychiatrist with expertise in the field of trauma, to validate the diagnosis.

Study exclusion criteria will be active malignancy; severe substance use disorders at baseline, current manic episode or psychotic disorders at baseline, serious suicidal ideation at baseline, severe or unstable physical disorders or major cognitive deficits at baseline; a history of HBOT, for any reason, prior to study enrollment; chest pathology incompatible with pressure changes (including active asthma); inner ear disease; claustrophobia; the inability to perform an awake brain MRI test; previous neurologic conditions (eg. History of traumatic brain injury, epilepsy, neuromuscular diseases, metabolic diseases, etc.); a brain tumor; skull base fractures; a history of neurosurgery and the inability to provide informed consent.

Study protocol:

After recruitment, participants will be randomized to one of two study groups. A treatment group will proceed to a course of HBOT, while the participants in the control group will continue to receive the care they received prior to enrolment in the study. After 3 months of follow up, the participants of both groups will be evaluated a second time, and will be offered to switch to the other group for an additional 3 month period.

HBOT protocol: a total of 60 daily HBOT sessions will be administrated 5 days per week,90 minutes exposure to 100% oxygen at 2 ATA, with 5-minute air breaks every 20 minutes.

All patients will be analyzed at baseline, after the first period of HBOT or standard care, and after the crossover period.

Study end points:

Primary end point

PTSD symptoms (CAPS)

Secondary end points

1.PTSD questionnaires:

2. sleep disorders and pain

Global Pain Scale (GPS) [45].

Medical Outcome Sleep Scale (MOS) [46]

3. Quality of life questionnaires:

Patient global impression of change [47]

SF-36 [48]

Quality of life will be evaluated by the EQ-5D

4. Diary for daily documentation of symptoms

5. Cognitive function

Cognitive function will be evaluated Mindstreams Cognitive Health Assessment (Mindstreams).

5.1 Mindstreams

This assessment comprises several cognitive tests that evaluate various aspects of brain capabilities. In the current study, we will establish a cognitive index based on scores of the 6 cognitive tests listed below, which have been shown to be relevant for mild TBI. For a detailed description of all the cognitive tests in Mindstreams Cognitive Health Assessment see [49]. The 6 tests are:

1. Verbal memory: Ten pairs of words are presented, followed by a recognition test in which the first word of a previously presented pair appears together with a list of four

words from which the participant chooses the other member of the pair. There are four immediate repetitions and one delayed repetition after 10 minutes.

2. Non-verbal memory. Eight pictures of simple geometric objects are presented, followed by a recognition test in which four versions of each object are presented, each oriented in a different direction. There are four immediate repetitions and one delayed repetition after 10 minutes.

3. Go–No-Go test. In this continuous performance test, a colored square (red, green, white or blue) appears randomly on the center of the screen. The participant is asked to respond quickly, only to red squares, by pressing the mouse button; and not to react to the presentation of any other colored square.

4. Stroop test. Timed test of response inhibition modified from the Stroop paper-based test. In the first phase, participants choose a colored square matching the color of a general word (for example, the word "Cat" appears in red letters; the participant must choose the red square of two colored squares in the following screen). In the next phase (termed the Choice Reaction Time test), the task is to choose the colored square matching the name of the color presented in white letter–color. In the final (Stroop interference) phase, participants are asked to choose the colored square that matches the color and not the meaning of a former color-naming word, presented in an incongruent color (for example, the word "RED" appears in green letters, the patient is asked to choose the color green and not red, a task requiring the ability to inhibit an automatic response to the meaning of the word).

5. Staged information processing test. Timed test requiring a reaction based on solving simple arithmetic problems (pressing the right/left mouse button if the answer is higher/lower than 4, respectively), with three levels of information processing load (single-digit, two-digit addition/subtraction and threedigit addition/ subtraction problems), each containing three speed levels (3, 2, and 1 second for the presentation of the stimuli).

6. Catch game. A test of motor planning that requires participants to “catch” a falling object on a computer screen by moving a paddle horizontally.

To assign scores, Mindstreams data will be uploaded to the NeuroTrax central server. Outcome parameters will be calculated using custom software blind to diagnosis or the testing site. To minimize differences related to age and education, each outcome parameter will be normalized and fit to an IQ-like scale (mean=100, STD=15), according to participants’ age and education. We note that the scores will be evaluated according to normative data from cognitively healthy individuals, collected in controlled research studies that were conducted at more than 10 clinical sites [50].

6. Brain imaging

Brain imaging will include 2 types of imaging: perfusion magnetic resonance imaging (MRI) + diffusion tensor imaging (DTI), including resting state functional MRI and brain single photon emission computed tomography (SPECT).

6.1 Perfusion MRI+DTI

The MRI protocol includes the following sequences: Axial T1, T2, FLAIR, Axial Diffusion weighted, Axial T1-PostGad, DSC, DCE, DTI, FLAIR,T1, T2 and SWI.

MRI scan sequence parameters:

DSC: 50 T2*-weighted gradient-echo echo planar imaging (EPI) volumes will be acquired, 2 repetitions before a bolus injection of Gadolinium-DTPA (Gd-DTPA), 48 repetitions after injection of Gd-DTPA. Sequence parameters: TR=2,300 ms, TE= 40ms, flip angle = 30°, voxel size = 1.8 x1.8, Matrix = 128x128, No. of slices = 25, Slice thickness = 3.9 mm.

DCE: three T1 weighted Fast Low Angle SHot (FLASH) volumes will be acquired with different flip angles (2, 7 and 15 degrees), followed by 70 dynamic T1 weighted FLASH volumes with flip angles of 15 degrees after a bolus injection of Gd-DTPA. Sequence parameters: before injection: TR=4.09, after injection - TR=4.86 ms, TE= 1.76 ms, Voxel size = 1.5x1.5, Matrix = 192X192, No. of slices = 26, Slice thickness = 3.5mm.

DTI: 30 diffusion weighted images will be scanned with various gradient directions (b=1000) and one volume without diffusion weighting, with the following parameters: TR=10,300 ms, TE=89 ms, Voxel size = 1.8X1.8, Matrix = 128 X 128, No. of slices = 63, Slice thickness = 2.2mm

Resting state fMRI(rsfMRI or R-fMRI)- a method of functional brain imaging that can be used to evaluate regional interactions that occur when a participant is not performing an explicit task. This resting brain activity is observed by means of changes in blood flow in the brain, which creates what is referred to as a blood-oxygen-level dependent (BOLD) signal that can be measured using functional MRI (fMRI).

6.2 Brain SPECT

SPECT will be conducted with 925–1,110 MBq (25–30 mCi) of technetium-99m-methyl-cysteinate-dimmer (Tc-99m-ECD) at 40–60 min post injection, using a dual

detector gamma camera (ECAM or Symbia T, Siemens Medical Systems) equipped with high resolution collimators. Data will be acquired in 3-degree steps and reconstructed iteratively by the Chang method (μ=0.12/cm) attenuation correction.

Regional CBF changes will be analyzed by fusing pre- and post-treatment studies that were normalized to median brain activity. SPECT images will be reoriented into Talairach space using NeuroGam (Segami Corporation) for identification of Brodmann cortical areas, and to compute the mean perfusion in each Brodmann area.

7. Brain Network Activation (BNA™) Analysis

The Brain Network Activation (BNA™) Analysis System is a software only device that utilizes advanced algorithms to analyze the brain network activity of the brain from the recorded electroencephalograph (EEG) data. The BNA Analysis system provides both qualitative network maps of activity, as well as quantitative scores that can be used as neuromarkers of disease.

Event Related Potentials (ERPs), which are temporal reflections of neural mass electrical activity of cells in specific regions of the brain, and which occur in response to stimuli, may offer such a method, as they provide a noninvasive and portable measure of brain function. ERPs provide excellent temporal information, but their spatial resolution has traditionally been limited. However, by means of high-density EEG recording, spatial resolution of ERPs is improved significantly [51-54]. The paradigm for the current study will combine neurophysiological knowledge with mathematical signal processing and pattern recognition methods (BNA) to temporally and spatially map brain function, connectivity and synchronization.

The following parameters will be evaluated by BNA technology:

Visual GO/NOGO - The Go-Nogo paradigm is among the most well-established tests of response inhibition to perceptual stimuli. The task involves sensory processing, motor activity (a response) and response inhibition. Accordingly, participants are presented a series of alphabetical letters and are required to react according to the letters. Letters are presented every 2 seconds on average. The letters B, C, D, E, F and G comprise 80% of the stimuli, and provide the Go cues ("Sensory”), to which participants are asked to respond as quickly as possible (“Motor”); 20% of the stimuli are an “X”, the No-Go cue ("Response inhibition”), which requires inhibition of response. Stimuli are presented on the center of a black background monitor for 150 ms, and are located between two vertical white lines that remain constant throughout the task. The inter stimulus interval varies randomly between 1000 ms and 2600 ms,

with increments of 250 ms. The total number of trials is 400. Task duration is ~14 minutes.

Auditory Oddball - The auditory oddball task is a classic EEG paradigm that has been studied extensively and used in a variety of neurological patient populations. The task involves executive functions, attention and memory processes. Participants hear a series of auditory tones and are required to react according to them. Tones are presented once every 1.5 seconds on average. A total of 80% of the sounds ("Sensory”) are 2000 Hz tones. A total of 10% of the sounds ("Memory") are 1000 Hz tones to which participants respond by pressing a button. The remaining 10% of sounds ("Attention") are complex sounds.

Sensory evoked potential - Contact heat stimuli are delivered to the right, dominant proximal volar forearm using a round thermode of 572.5 mm2 (PATHWAY, Medoc Ltd., Ramat-Yishai, Israel) in four-block heat stimulation sessions to produce contact heat evoked potential (CHEP). Each CHEP stimulus trial begins with the thermode applied to the skin, followed by triggering of the heat stimulus and ending with a beep, which prompts participants to rate the pain produced by the heat stimulus. This thermode application is repeated in a clockwise manner on the designated area of the forearm. On Visit 1, participants undergo a temperature-determination protocol, in which they receive three heat stimuli of the same temperature, ranging from 38°C to 52°C. They are instructed to rate each stimulus on a numeric rating scale (NRS), in which 0 indicates the absence of pain and 10 indicates the worst imaginable pain. The two temperatures that participants rate as 3 and 6 on the NRS are chosen as low and high-temperatures, respectively, and are used during the proceeding pre- and post HD-tDCS EEG recordings. The first two blocks of heat stimuli use the low-temperature that was determined (NRS rating of 3) for each individual and are separated by a 5-minute interval. The following two blocks of heat stimuli use the high-temperature that was determined (NRS rating of 6) for each individual, and are also separated by a 5-minute break. The low and high-temperature blocks are further separated by a 15-minute interval. Twenty stimuli (trials) are performed per block and the inter-stimulus interval ranges from 8 to 13 sec.

Resting States- EEG will be recorded at rest (no cognitive task), several minutes after eyes are open, and several minutes after eyes are closed (between 2 and 5 minutes). The aim of the resting EEG recording is to extract patterns of the default mode network (DMN). The DMN comprises several brain regions, and has been shown, in fMRI studies, to reduce activation during task execution. The DMN has recently been studied also in EEG signals (Laufs et al. 2003).

Physiological evaluation of brain functionality using TMS-EEG examination:
This examination includes non-invasive safe brain monitoring and stimulation for the assessment of changes in brain functionality and connectivity. The procedure includes placement of EEG electrodes on an individual's head and measurement of the TMS-evoked electrophysiological response in the various brain regions. The examination takes about 30 minutes.

8. Physical activity and sleep

5.1 Monitoring 24/7 physical activity- Daily physical activity and sleep will be objectively tracked by Polaris watch technology. The Polaris watch will be also wired at night for measurements of the time asleep, restlessness and time awake,

9. Immune system

9.1 Inflammatory cytokines

Blood Tests will include: IL-1, IL-6, Tumor necrosis factor-alpha, CRP.

10. Endocrine system

Blood Tests will include: free cortisol, ACTH, TSH

Ref

1. Archambeau, O.G., J.D. Elhai, and C.B. Frueh, *Definition of psychological trauma and threshold for functional impairment in PTSD.* J Clin Psychiatry, 2011. **72**(3): p. 416-7.
2. Christianson, S.A., *Emotional stress and eyewitness memory: a critical review.* Psychol Bull, 1992. **112**(2): p. 284-309.
3. Gold, P.E. and R.B. Van Buskirk, *Facilitation of time-dependent memory processes with posttrial epinephrine injections.* Behav Biol, 1975. **13**(2): p. 145-53.
4. Sandi, C. and S.P. Rose, *Corticosterone enhances long-term retention in one-day-old chicks trained in a weak passive avoidance learning paradigm.* Brain Res, 1994. **647**(1): p. 106-12.
5. Morena, M., et al., *Effects of ketamine, dexmedetomidine and propofol anesthesia on emotional memory consolidation in rats: Consequences for the development of post-traumatic stress disorder.* Behav Brain Res, 2017. **329**: p. 215-220.
6. Wan, J., et al., *Single-prolonged stress induce different change in the cell organelle of the hippocampal cells: A study of ultrastructure.* Acta Histochem, 2016. **118**(1): p. 10-9.
7. Rasmusson, A.M., et al., *Low baseline and yohimbine-stimulated plasma neuropeptide Y (NPY) levels in combat-related PTSD.* Biol Psychiatry, 2000. **47**(6): p. 526-39.
8. Perry, B.D., E.L. Giller, Jr., and S.M. Southwick, *Altered platelet alpha 2-adrenergic binding sites in posttraumatic stress disorder.* Am J Psychiatry, 1987. **144**(11): p. 1511-2.
9. Maes, M., et al., *Serotonergic and noradrenergic markers of post-traumatic stress disorder with and without major depression.* Neuropsychopharmacology, 1999. **20**(2): p. 188-97.
10. van Zuiden, M., et al., *Pre-existing high glucocorticoid receptor number predicting development of posttraumatic stress symptoms after military deployment.* Am J Psychiatry, 2011. **168**(1): p. 89-96.
11. McGaugh, J.L., *Memory--a century of consolidation.* Science, 2000. **287**(5451): p. 248-51.
12. Holbrook, T.L., et al., *Morphine use after combat injury in Iraq and post-traumatic stress disorder.* N Engl J Med, 2010. **362**(2): p. 110-7.
13. Yehuda, R., *Post-traumatic stress disorder.* N Engl J Med, 2002. **346**(2): p. 108-14.
14. Charney, D.S., *Psychobiological mechanisms of resilience and vulnerability: implications for successful adaptation to extreme stress.* Am J Psychiatry, 2004. **161**(2): p. 195-216.
15. Pitman, R.K., et al., *Biological studies of post-traumatic stress disorder.* Nat Rev Neurosci, 2012. **13**(11): p. 769-87.
16. Bonne, O., et al., *Resting regional cerebral perfusion in recent posttraumatic stress disorder.* Biol Psychiatry, 2003. **54**(10): p. 1077-86.
17. Lucey, J.V., et al., *Brain blood flow in anxiety disorders. OCD, panic disorder with agoraphobia, and post-traumatic stress disorder on 99mTcHMPAO single photon emission tomography (SPET).* Br J Psychiatry, 1997. **171**: p. 346-50.
18. Zhe, X., et al., *Decreased Regional Cerebral Perfusion at Resting State in Acute Posttraumatic Stress Disorder Resulting From a Single, Prolonged Stress Event.* Acad Radiol, 2016. **23**(9): p. 1083-90.

19. Yehuda, R., et al., *Post-traumatic stress disorder.* Nat Rev Dis Primers, 2015. **1**: p. 15057.
20. Shin, L.M. and I. Liberzon, *The neurocircuitry of fear, stress, and anxiety disorders.* Neuropsychopharmacology, 2010. **35**(1): p. 169-91.
21. Vermetten, E., et al., *Positron tomographic emission study of olfactory induced emotional recall in veterans with and without combat-related posttraumatic stress disorder.* Psychopharmacol Bull, 2007. **40**(1): p. 8-30.
22. Battle, D.E., *Diagnostic and Statistical Manual of Mental Disorders (DSM).* Codas, 2013. **25**(2): p. 191-2.
23. Neylan, T.C., et al., *Neuroendocrine regulation of sleep disturbances in PTSD.* Ann N Y Acad Sci, 2006. **1071**: p. 203-15.
24. Kessler, R.C. and P.S. Wang, *The descriptive epidemiology of commonly occurring mental disorders in the United States.* Annu Rev Public Health, 2008. **29**: p. 115-29.
25. Lohr, J.B., et al., *Is Post-Traumatic Stress Disorder Associated with Premature Senescence? A Review of the Literature.* Am J Geriatr Psychiatry, 2015. **23**(7): p. 709-25.
26. Boussi-Gross, R., et al., *Hyperbaric oxygen therapy can improve post concussion syndrome years after mild traumatic brain injury - randomized prospective trial.* PLoS One, 2013. **8**(11): p. e79995.
27. Efrati, S., et al., *Hyperbaric oxygen induces late neuroplasticity in post stroke patients--randomized, prospective trial.* PLoS One, 2013. **8**(1): p. e53716.
28. Hadanny, A. and S. Efrati, *Treatment of persistent post-concussion syndrome due to mild traumatic brain injury: current status and future directions.* Expert review of neurotherapeutics, 2016. **16**(8): p. 875-87.
29. Tal, S., et al., *Hyperbaric oxygen may induce angiogenesis in patients suffering from prolonged post-concussion syndrome due to traumatic brain injury.* Restorative neurology and neuroscience, 2015. **33**(6): p. 943-51.
30. Hadanny, A., et al., *Hyperbaric oxygen can induce neuroplasticity and improve cognitive functions of patients suffering from anoxic brain damage.* Restorative neurology and neuroscience, 2015. **33**(4): p. 471-86.
31. Boussi-Gross, R., et al., *Improvement of memory impairments in poststroke patients by hyperbaric oxygen therapy.* Neuropsychology, 2015. **29**(4): p. 610-21.
32. Efrati, S. and E. Ben-Jacob, *Reflections on the neurotherapeutic effects of hyperbaric oxygen.* Expert review of neurotherapeutics, 2014. **14**(3): p. 233-6.
33. Chang, C.C., et al., *Damage of white matter tract correlated with neuropsychological deficits in carbon monoxide intoxication after hyperbaric oxygen therapy.* J Neurotrauma, 2009. **26**(8): p. 1263-70.
34. Vilela, D.S., P.R. Lazarini, and C.F. Da Silva, *Effects of hyperbaric oxygen therapy on facial nerve regeneration.* Acta Otolaryngol, 2008. **128**(9): p. 1048-52.
35. Haapaniemi, T., et al., *Hyperbaric oxygen treatment enhances regeneration of the rat sciatic nerve.* Exp Neurol, 1998. **149**(2): p. 433-8.
36. Chen, Z., et al., *Visual pathway lesion and its development during hyperbaric oxygen treatment: a bold- fMRI and DTI study.* J Magn Reson Imaging, 2010. **31**(5): p. 1054-60.
37. Neubauer, R.A. and P. James, *Cerebral oxygenation and the recoverable brain.* Neurol Res, 1998. **20 Suppl 1**: p. S33-6.

38. Eve, D.J., et al., *Hyperbaric oxygen therapy as a potential treatment for post-traumatic stress disorder associated with traumatic brain injury.* Neuropsychiatr Dis Treat, 2016. **12**: p. 2689-2705.
39. Tal, S., et al., *Hyperbaric oxygen may induce angiogenesis in patients suffering from prolonged post-concussion syndrome due to traumatic brain injury.* Restor Neurol Neurosci, 2015. **33**(6): p. 943-51.
40. Efrati, S. and E. Ben-Jacob, *Reflections on the neurotherapeutic effects of hyperbaric oxygen.* Expert Rev Neurother, 2014. **14**(3): p. 233-6.
41. Hoge, C.W., et al., *Mild traumatic brain injury in U.S. Soldiers returning from Iraq.* N Engl J Med, 2008. **358**(5): p. 453-63.
42. Bryant, R.A., et al., *The psychiatric sequelae of traumatic injury.* Am J Psychiatry, 2010. **167**(3): p. 312-20.
43. Cifu, D.X., et al., *The effect of hyperbaric oxygen on persistent postconcussion symptoms.* J Head Trauma Rehabil, 2014. **29**(1): p. 11-20.
44. Harch, P.G., et al., *A phase I study of low-pressure hyperbaric oxygen therapy for blast-induced post-concussion syndrome and post-traumatic stress disorder.* J Neurotrauma, 2012. **29**(1): p. 168-85.
45. Tory McJunkin, M., *Reliability and validity of the Global Pain Scale with chronic pain sufferers.* Pain physician, 2011. **14**: p. 61-70.
46. Williams, D.A. and L.M. Arnold, *Measures of fibromyalgia: Fibromyalgia Impact Questionnaire (FIQ), Brief Pain Inventory (BPI), Multidimensional Fatigue Inventory (MFI-20), Medical Outcomes Study (MOS) Sleep Scale, and Multiple Ability Self-Report Questionnaire (MASQ).* Arthritis care & research, 2011. **63**(S11): p. S86-S97.
47. Ferguson, L. and J. Scheman, *Patient global impression of change scores within the context of a chronic pain rehabilitation program.* The Journal of Pain, 2009. **10**(4): p. S73.
48. Neumann, L., A. Berzak, and D. Buskila. *Measuring health status in Israeli patients with fibromyalgiasyndrome and widespread pain and healthy individuals: Utility of the Short Form 36-item health survey (SF-36).* in *Seminars in arthritis and rheumatism*. 2000. Elsevier.
49. Doniger, G.M. *Mindstreams Computerized Cognitive Tests: Test Descriptions. Available: http://www.mirror.upsite.co.il/uploaded/files/1383_e7d7d3d98c924f036d3123733419149d.pdf. Accessed 05 July 2013*. 2007 [cited 2013 05 July]; Available from: http://www.mirror.upsite.co.il/uploaded/files/1383_e7d7d3d98c924f036d3123733419149d.pdf.
50. Doniger, G.M. *Guide to MindStreams Normative Data. Available: http://www.mirror.upsite.co.il/uploaded/files/1383_b44d4786c91058be301cb09a94ba70f4.pdf. Accessed 05 July 2013*. 2012; Available from: http://www.mirror.upsite.co.il/uploaded/files/1383_b44d4786c91058be301cb09a94ba70f4.pdf.
51. Castillo-Saavedra, L., et al., *Clinically Effective Treatment of Fibromyalgia Pain With High-Definition Transcranial Direct Current Stimulation: Phase II Open-Label Dose Optimization.* The journal of pain : official journal of the American Pain Society, 2016. **17**(1): p. 14-26.

52. Reches, A., et al., *A novel electroencephalography-based tool for objective assessment of network dynamics activated by nociceptive stimuli.* European journal of pain, 2016. **20**(2): p. 250-62.
53. Reches, A., et al., *Preliminary investigation of Brain Network Activation (BNA) and its clinical utility in sport-related concussion.* Brain injury, 2017: p. 1-10.
54. Kiefer, A.W., et al., *Brain Network Activation as a Novel Biomarker for the Return-to-Play Pathway Following Sport-Related Brain Injury.* Frontiers in neurology, 2015. **6**: p. 243.
