# Supplementary material for: Hyperbaric oxygen therapy improves symptoms, brain’s microstructure and functionality in veterans with treatment resistant post-traumatic stress disorder: A prospective, randomized, controlled trial
Source: PLoS One. 2022 Feb 22;17(2):e0264161. doi: 10.1371/journal.pone.0264161 (PMC8863239; doi:10.1371/journal.pone.0264161)
Supplement: Attachment — Submitted filename: PONE-D-20-34405 rebuttal letter.docx [file pone.0264161.s009.docx]

To: Miquel Vall-llosera Camps

Senior Editor, PLOS ONE

PONE-D-20-34405: "Hyperbaric oxygen therapy improves symptoms, brain's microstructure and functionality in veterans with treatment resistant post-traumatic stress disorder: a prospective, randomized, controlled trial."

Dear Miquel Vall-llosera Camps

We thank you and the reviewers for your thorough review of our manuscript. We have addressed to each of the suggested comments. We hope that you will find the revised version suitable for publication.

Best regards,

Keren Doenyas-Barak

Head of PTSD program

Sagol center for hyperbaric medicine

Shamir Medical Center, Zerifin, Israel

**Reply to Review Comments to the Author**

**Reviewer #1:**

This is an excellent paper on a important topic. It is well written and the conclusions are sound. The groups are clearly define, as well as the diagnosis and treatment methods. It is also easy to understand. It provides a new way to address PTSD by optimizing brain function.

**Reply:** we thank the reviewer for his support.

**Reviewer #2:**

I thank the authors for the opportunity to review the reporting of their important research study. While I found the manuscript generally well written and concise, clarification of a couple of things could improve the quality and scientific impact of the paper in the field.

**Comment 1**: “On page 11, it’s not clear why prescribed cannabis (mean grams/month) is reported differently than the other prescribed medication (number of patients (% of group)). The reader is left wondering what number and percentage in each group were using prescribed cannabis during the study”.

**Reply:** *The number and percentage of patients using cannabis in each group were added to table 1.*

**Comment 2:** “On page 16, line 28, the authors write: "One of the interesting findings in the current study, was the surfacing of unaccusable memories in half of the patients from the HBOT group." If the adjective "unaccusable" (not charged with wrongdoing) used here was intended to convey psychological meaning regarding the nature of previously "inaccessible" memories, then "unaccusable memories" should be operationally defined here.”

**Reply:** Thank you for the comment. The term *“unaccusable memories”* was replaced by the more appropriate term *“inaccessible memories”.*

**Comment 3**: “since this phenomenon occurred in half those receiving treatment (n=7?) and this has only been reported once previously in the literature, this could be an important psychological predicter of therapeutic response that deserves further quantitative and qualitative analysis and discussion, especially in regards to maximizing psychological interventions for PTSD. For example, quantitatively, did those who experienced these repressed memories differ on clinical outcome measures from those who didn’t experience them within the HBOT treatment group? Were any time course patterns observed in regards to when these repressed memories began to emerge following the start of treatment? For example, in the previous fibromyalgia HBOT study cited by the authors, time course information was only provided for two of the nine patients, with reemergence of repressed memories occurring between HBOT days 17 and 34 in one and HBOT days 37 and 44 in the second patient. It would also be of interest to provide more information regarding the qualitative nature of these repressed memories and how they resolved psychotherapeutically. For example, the authors should consider providing a brief and concise clinical vignette on one or more of these 7 PTSD patients as illustrative examples as was provided in the previous fibromyalgia HBOT study.”
**Reply:** Additional data regarding the time and pattern of the reported memories was added to the results section of the revised manuscript.
The surfacing of the repressed memories was not anticipated prior to the study so no intentional questioning and monitoring of this phenomenon was done as part of the study protocol. Since it might be possible that other participants had recovery of memories and did not report it, we cannot use the current data for additional conclusions on the psychological meaning of this effect.

we agree that the surfacing memories has the potential to serve as a psychological predictor of therapeutic response and it indeed deserves further quantitative and qualitative analysis. Accordingly, our next study will include in depth evaluation and actively monitored of all participants with multiple aspects related to their memory.

**Reviewer #3:**

**Comment 1**: “Sample size determination is not acceptable. Need to add more details, e.g. effect size.

**Replay**: Data related to sample size calculation based on effect size was added to the Statistical analysis - Sample size section.

**Comment 2**: “Data analysis section needs more work. Be consistent with the statistical terms. Just use “Repeated ANOVA” instead of “within-subject RM ANOVA ” or “mixed design RM ANOVA”. Two names were used for p value correction also: “sequence Hochberg” and “FDR (Figure 4)”, use one.

**Replay**: Thank you for this comment. The ANOVA and the multiple comparisons methods were not described clearly enough. A mixed-model repeated-measure ANOVA was performed to compare post-treatment and pre-treatment data. The model included time, group and the group-by-time interaction (detailed results are presented in Supporting Tables SI-1, SI-2).

Brain imaging parametric maps were corrected using the Benjamini–Hochberg False Discovery Rate (FDR) method (ref. 22 in the revised manuscript). Group-by-time interaction imaging analysis was corrected using the sequential Hochberg correction (ref. 23 in the revised manuscript).

The data analysis section was revised accordingly.

**Comment 3**: “What method was used if the data does not follow normal distribution?”
**Replay**: In this study, all continuous data were tested by the Kolmogorov-Smirnov test and found to be normally distributed.

**Comment 4**: “What is the “net effect size”?”

**Replay**: Net effect size is the relative Cohen’s d effect size, defined as the improvement from baseline after HBOT minus control three months improvement divided by the pooled standard deviation (SD) of the composite score. Positive effect sizes indicate improvement. An explanation was added to the statistical analysis section.

**Comment 5**: “Page 9 line 6 the sentence “univariate analyses were performed … significant variables” can be omitted. It does not seem that you performed univariable or multivariable analyses.”

**Replay**: This was referred to the categorical data, and corrected in the revised manuscript.

**Comment 6**: “Results - For RM ANOVA, it is not meaningful to show a significant interaction. For each outcome, better show at least three p values and mean differences (with 95% confidence intervals) for the following tests: 1) pre-post test within the treatment group, 2) pre-post test within the control group, 3) a comparison of pre-post change between the treatment and control group. The p value and mean difference (95%) for pre-post change between the two groups should be shown in Table 2 and 3.”

**Replay**: The group-by-time interaction ANOVA method is a common method for demonstrating outcomes between groups controlling for time. However, we also added p-values and mean differences with 95% confidence intervals, as suggested (Tables 2 and 3).

**Comment 7**: “Need to test normality for RM ANOVA. Bonferroni not mentioned in method but not in the results.”

**Replay**: Indeed, RM ANOVA model is valid for normally distributed data. All continuous data in this study were normally distributed.

*Non-imaging data was corrected for multiple comparisons using the Bonferroni correction. Marked and added to Tables 2 and 3.*

**Comment 8**: “Page 17 line 6 “affect” instead of “effect””

**Replay**: Done.

**Additional requirements:**

1. We removed the funding statement from the acknowledgement. Please assist us with entering following statement to the online submission form:
   "The study was funded by the Research Fund of Shamir Medical Center, Israel."
2. Conflict of interests:
   Please assist us with entering following statement to the online submission form.
   AH and ES works for AVIV scientific. SE is a shareholder in AVIV scientific
3. Minimal data set:
   A table with patients’ data was added as supporting Information.
