# Supplementary material for: Hyperbaric oxygen therapy improves symptoms, brain’s microstructure and functionality in veterans with treatment resistant post-traumatic stress disorder: A prospective, randomized, controlled trial
Source: PLoS One. 2022 Feb 22;17(2):e0264161. doi: 10.1371/journal.pone.0264161 (PMC8863239; doi:10.1371/journal.pone.0264161)
Supplement: Attachment — Submitted filename: rebuttal letter.docx [file pone.0264161.s010.docx]

Dear Prof. Burak Yulug

We thank you for your review of our manuscript.

We have addressed to the suggested comment. We hope that you will find the revised version suitable for publication.

Best regards,

Keren Doenyas-Barak

Head of PTSD program

Sagol center for hyperbaric medicine

Shamir Medical Center, Zerifin, Israel

**Journal Requirements:**

Please review your reference list to ensure that it is complete and correct. If you have cited papers that have been retracted, please include the rationale for doing so in the manuscript text, or remove these references and replace them with relevant current references. Any changes to the reference list should be mentioned in the rebuttal letter that accompanies your revised manuscript. If you need to cite a retracted article, indicate the article’s retracted status in the References list and also include a citation and full reference for the retraction notice.

*Reply*: Reference no 21 was added to the revised manuscript and the list was updated accordingly.

We reviewed the reference list to make sure that it is complete and correct.

**Editor Comment:**

This is an interesting paper indicating the therapeutic role of HBOT on PTSD associated with relevant changes in the brain network and microstructure. Although the authors responded well to the reviewers' recommendation, I think that more detail regarding the cognitive baseline data of the patients should be included in the main text. For instance, were all patients cognitively homogeneously distributed in this respect, which could be critical in affecting both the clinical and imaging data. More relevant data regarding the questions should be provided in the main text since cognitive status could be game-changer in such clinical neuroimaging studies: What is the dementia/cognitive impairment threshold of MindStream. Why the authors did not use MMSE which is widely used for dementia exclusion.

*Reply*: We thank you for your review of our manuscript.

The Neurotrax tool box was chosen for baseline cognitive evaluation, as it is fully computerized test that was validated also among cognitively healthy subjects (1-2). The cognitive scores are presented as normalized scores according to age and education groups, on an IQ-style scale, where 100 represents the mean normalized score and one standard deviation equals to 15 points.

The baseline global cognitive score was on the normal range expected for the patients’ age and gender, 99.4±6.2 and 98.5±8.7 in the HBOT and control group respectively, p=0.75.

We have added the requested data regarding baseline cognitive score to the revised manuscript.

1. Doniger, G.M., *Description of the MindStreams® Normative Database.* 2008.

2. Doniger, G.M. and E.S. Simon, *Construct validity of mindstreams: comparison with paper-based tests.* Bellaire, TX: NeuroTrax Corp, 2013.
